# The effect of physical activity on life satisfaction among sports science students: a moderated mediation model of resilience and mindfulness

**DOI:** 10.3389/fpsyg.2025.1682469

**Published:** 2025-10-10

**Authors:** Emrah Secer, Zulbiye Kacay, Nuh Osman Yildiz, Laurentiu-Gabriel Talaghir, Dumitru Marius Cosoreanu, Cristina Corina Bentea

**Affiliations:** ^1^Faculty of Sport Sciences, Erzincan Binali Yıldırım University, Erzincan, Türkiye; ^2^Faculty of Sport Sciences, Çanakkale Onsekiz Mart University, Çanakkale, Türkiye; ^3^Faculty of Sport Sciences, Bolu Abant İzzet Baysal University, Bolu, Türkiye; ^4^Faculty of Physical Education and Sport, Dunarea de Jos University of Galati, Galati, Romania

**Keywords:** life satisfaction, mindfulness, moderated mediation, physical activity, psychological resilience

## Abstract

**Background:**

This study examines the effect of physical activity (PA) on life satisfaction (LS), focusing on the mediating role of psychological resilience (PR) and the moderating role of mindfulness (MF).

**Method:**

Participants were 363 sports science students (240 male and 123 female). Data were collected through International Physical Activity Questionnaire-Short Form, the Life Satisfaction Scale, the Psychological Resilience Scale-Short Form and the Mindfulness Scale. The measurement model was tested using confirmatory factor analysis (CFA) in AMOS 21 and hypothesis were analyzed using Hayes’ (2022) Process Macro models (4, 1, and 7).

**Results:**

The mediation effect (Model 4) indicated that the indirect effect of PA on LS through PR was significant. The moderation analysis (Model 1) showed that mindfulness significantly moderated the relationship between PA and PR. Finally, the moderated mediation model (Model 7) confirmed that the indirect effect of PA on LS via PR was stronger at higher levels of mindfulness. Although the effect size was small, it highlights the practical importance of integrating mindfulness in resilience pathways.

**Conclusion:**

These findings suggest that physical activity contributes to life satisfaction by enhancing resilience, and that this indirect effect is conditioned by mindfulness. These findings point to the potential value of resilience-building and mindfulness-based programs in educational and psychological practice. However, as the study sample consisted of sports science students from a single faculty, the results should be interpreted with caution regarding generalizability.

## 1 Introduction

Life satisfaction is a multidimensional construct shaped not only by external conditions but also by individual resources such as physical activity, psychological resilience and mindfulness for university students, life satisfaction is influenced by academic achievement, social relationships, physical health and emotional flexibility and also by family resource allocation and time management (Hong et al., 2024). Physical activity contributes not only to physical health but also to psychological functioning, serving as a lifestyle factor that supports stress regulation, self-perceptions and well-being. However, the relationship between physical activity and life satisfaction is not linear; it is shaped by meditating mechanisms such as resilience and moderating mechanisms such as mindfulness, which reflect the individual’s coping capacity and mental resources. In this context, psychological resilience is defined as an individual’s ability to demonstrate flexibility, recovery and adaptation in the face of challenges. Mindfulness, on the other hand, is a mental attitude that enables an individual to focus on momentary experiences without judgment, thereby strengthening their internal resources. Therefore, while the contribution of physical activity to life satisfaction can increase through an individual’s level of psychological resilience, the strength of this relationship may vary depending on the individual’s level of mindfulness. This multi-layered structure requires more in-depth analyses to understand the individual’s mind–body balance. In addition, demographic and family structure variables such as children’s gender have been linked to long-term well-being outcomes (Gao et al., 2025). This study aims to address this need by examining the relationships between physical activity, psychological resilience, mindfulness and life satisfaction on both theoretical and empirical levels.

### 1.1 Physical activity and life satisfaction

According to the World Health Organization (2020), physical activity encompasses planned, structured and repetitive movements that increase energy expenditure. Exercise, sports and recreational activities that are part of daily life are also considered core components of physical activity (Piercy et al., 2018). Beyond its physiological benefits, a growing body of evidence indicates that physical activity is positively associated with subjective well-being and life satisfaction (Bize et al., 2007; Carriedo et al., 2020; Ginis, 2025; Marcos-Pardo et al., 2023; Potoczny et al., 2025). Life satisfaction refers to an individual’s overall evaluation of their life as satisfying, meaningful and positive also constitutes an essential dimension of subjective well-being (Diener et al., 2018). An individual’s sense of satisfaction with their own life and their positive subjective evaluation are among the fundamental elements that define life satisfaction (Pavot and Diener, 2008). The relationship between physical activity and life satisfaction can be explained by multiple theoretical perspectives. Self-Determination Theory (SDT) argues that physical activity supports basic psychological needs of autonomy, competence, and relatedness, which in turn enhance intrinsic motivation and life satisfaction (Deci and Ryan, 2000; Teixeira et al., 2012). Flow Theory emphasizes that deeply engaging in physical activity generates optimal psychological states, contributing to well-being (Csíkszentmihályi, 1997; Swann et al., 2012). The mind–body perspective highlights neurobiological pathways, suggesting that physical activity reduces stress reactivity and increases endorphins and serotonin, thereby enhancing life satisfaction (Dishman et al., 2012; Mikkelsen et al., 2017). In addition, from a Social Learning Theory perspective, social support and observational learning may encourage engagement in physical activity, indirectly contributing to life satisfaction (Bandura, 1986). From a Positive Psychology perspective, physical activity is viewed as an activity that fosters positive affect and resilience leading to greater life satisfaction (Seligman, 2011; Fredrickson, 2013). Importantly, these perspectives should not be considered in isolation but as complementary: while SDT and Flow explain motivational and experiential aspects, mind–body and positive psychology highlight biological, emotional and social mechanisms. Together, these frameworks provide a multidimensional lens to understand how physical activity contributes to life satisfaction. Moreover, advanced computational approaches have been developed to predict happiness and life satisfaction, integrating psychological and social determinants (Wu et al., 2025b; Wu et al., 2025a).

### 1.2 The mediating role of psychological resilience

Psychological resilience is defined as an individual’s capacity to cope with stressful, challenging, and traumatic situations and is an essential component of psychological well-being (Connor and Davidson, 2003). This concept refers to an individual’s ability to demonstrate resilience in the face of adversity in their life and to achieve positive adaptation during this process (Smith et al., 2008; Yin et al., 2023). Psychological resilience refers to an individual’s capacity to develop effective coping strategies in the face of prolonged stress, difficulties or traumatic life events (Graber et al., 2015). This concept is highlighted as a competence that supports individuals in acting in harmony with their social environment and maintaining their life balance (Schwarz, 2018). Navrady et al. (2018) define psychological resilience as an individual’s ability to maintain or regain mental health. Preventive intervention programs that support psychological health play a crucial role in preventing the emergence of potential psychological problems and enhancing individuals’ well-being (McGovern et al., 2024). In this context, psycho-educational programs are widely used in preventive and development-oriented interventions developed for young people especially in school environments (Nisar et al., 2025). In recent years, psychological resilience has garnered increasing interest in both clinical and sports psychology and the effects of physical activity on psychological resilience have been supported by numerous studies (Fletcher and Sarkar, 2016). Physical activity is recognized as a crucial factor in enhancing psychological resilience and coping with stress. Regular physical activity helps regulate stress hormones (e.g., cortisol), enhances self-confidence and fosters psychological flexibility (Salmon, 2001). Studies supporting this situation also show that regular physical activity has a positive effect on psychological resilience levels in university students (Çakır et al., 2025; Peng et al., 2025; Seçer and Yıldızhan, 2020; Qiu et al., 2025). Resilience Theory emphasizes the interaction of environmental, individual and social factors in the development of psychological resilience (Masten, 2018). In this context, the social interaction, self-efficacy and emotional regulation processes provided by physical activity play a mediating role in increasing psychological resilience (Windle, 2011). Social learning theory also states that individuals can develop psychological resilience by observing positive behavior models through physical activity (Bandura, 1986). From a positive psychology perspective, physical activity promotes positive moods and optimism in individuals thereby laying the groundwork for psychological resilience (Fredrickson, 2013; Zautra et al., 2010). Additionally, stress management theories suggest that physical activity enhances psychological resilience by facilitating cognitive and emotional regulation in stressful situations (Lazarus and Folkman, 1984). As a result, the positive effects of physical activity on psychological resilience emerge as an essential mechanism in helping individuals achieve life satisfaction. Thus, resilience can be conceptualized as a psychological mechanism through which physical activity enhances life satisfaction by improving coping capacity, emotional regulation and optimism.

### 1.3 The moderating role of mindfulness

Mindfulness is defined as the state of being aware of one’s current experiences in an open and accepting manner without judgment (Henriksen et al., 2020; Kabat-Zinn, 2003). In current psychological literature, mindfulness is considered a crucial moderating factor that enhances individuals’ stress-coping skills and strengthens psychological resilience (Gu et al., 2015). Mindfulness practices combined with sports (Birrer et al., 2023; Piasecki et al., 2025) play a crucial role in reducing psychological issues such as anxiety and depression, while also supporting positive psychological states (Hölzel et al., 2011; Khoury et al., 2013). Mindfulness acts as a critical variable that determines the strength and direction of this relationship by regulating the effect of physical activity on psychological resilience (Creswell, 2017). Within the mind–body connection theory, mindfulness increases psychological resilience and enables individuals to cope more effectively with stress by recognizing and regulating bodily experiences (Garland et al., 2015a, 2015b). This suggests that individuals with high mindfulness tend to benefit more from the positive effects of physical activity. In addition, constructivist developmental theories suggest that mindfulness supports learning and development processes by regulating an individual’s cognitive and emotional processes (Langer, 2014; Vygotsky and Cole, 1978). From this perspective, mindfulness enables individuals to respond more harmoniously and flexibly to environmental and internal stimuli (Baer, 2015). Recent studies have revealed the positive effects of mindfulness on motivation, focus and performance in the context of sports and physical activity (Sappington and Longshore, 2015). Therefore, the moderating role of mindfulness is critical in understanding the effects of physical activity on psychological resilience and life satisfaction. These theoretical and empirical findings reveal that mindfulness functions as an essential regulatory variable in an individual’s psychological functioning and can shape the effects of physical activity on mental resilience. In this context, the present study considers psychological resilience as a mediator and mindfulness as a moderator to gain a deeper understanding of the effects of physical activity on life satisfaction.

The moderating effect of mindfulness suggests that the strength of the indirect relationship between physical activity and life satisfaction via resilience may vary by mindfulness level. Students with higher mindfulness are more likely to benefit from resilience-building effects of physical activity whereas those with lower mindfulness may show weaker associations.

While previous studies have generally focused on testing simple and direct relationships between these variables, the current study was conducted to gain a deeper understanding of social reality using a model that includes mediating and moderating variables, based on the limitations and inadequacies of this approach. By including mediating and moderating variables in the model, an attempt was made to reveal more qualitative mechanisms explaining the effects of physical activity on life satisfaction. The lack of previous studies addressing a comprehensive model covering these variables further highlights the original contribution and value of this research. Indeed, theoretical foundations also support this. Furthermore, the fact that the study was conducted in Türkiye, which has different socio-cultural dynamics, in a field dominated by Western literature, contributes to the cross-cultural generalizability of the literature, in addition to providing meaningful contributions to the local context.

Based on the theoretical framework, we formulated the following hypotheses:

*H1:* Physical activity indirectly and positively affects life satisfaction through psychological resilience.

*H2:* Mindfulness moderates the relationship between physical activity and psychological resilience such that the positive effect is stronger for students with high mindfulness.

*H3:* Mindfulness moderates the indirect effect of physical activity on life satisfaction through psychological resilience, such that the indirect effect is stronger for students with high mindfulness.

## 2 Method

### 2.1 Research model

**Figure 1:**
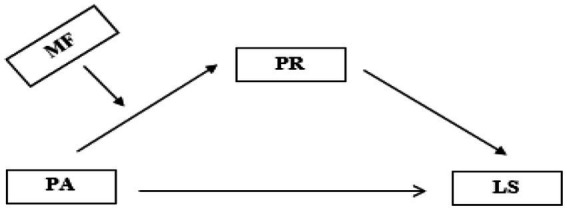
Research model.

This study investigates the effect of physical activity levels on life satisfaction among university students in the field of sports science. Additionally, the mediating role of psychological resilience and the moderating role of mindfulness in this effect are investigated. This study was conducted within the framework of the survey model which aims to explore the relationships between two or more variables and is a quantitative research method (Christensen et al., 2015). In recent years, a growing view in social science research has emerged that merely identifying relationships or effects is insufficient to explain the complexity of social phenomena. In this context, the need to focus on the conditions under which relationships arise (moderating effects) and the mechanisms through which these relationships are shaped (mediating effects) is emphasized. It is stated that such analyses are essential for deepening theoretical models and contributing to the field (Gürbüz, 2021). The theoretical model tested in the study is presented in Figure 1.

### 2.2 Research group

The power analysis for determining the sample size in the study was performed using the G*Power software based on a multiple regression model. In the analysis, it was calculated that a minimum of 215 participants were required, assuming a statistical power of 95% (1−*β* = 0.95), a significance level of 0.05 (*α* = 0.05) and a medium effect size (f^2^ = 0.10). However, considering Suresh and Chandrashekara's (2012) recommendation to increase the sample size by approximately 10% to account for potential data loss or missing data, the final sample consisted of 363 participants. Within this scope, 120 female students (age 18–46, 21.23 ± 3.110) and 243 male students (age 18–48, 21.56 ± 3.944) participated in the study.

The research group consists of participants enrolled in sports science faculties at universities in Türkiye, selected using criterion sampling, one of the purposeful sampling methods. The participant selection criteria are as follows: (1) being a student in a sports science faculty and (2) actively participating in sports activities. These criteria were determined to ensure that participants had the experience and characteristics appropriate for the research purpose. Although the sampling process was not random, participants who met the specified criteria were selected voluntarily. In this respect, the sample has characteristics of non-probability and convenience sampling. This limits the generalizability of the results to wider populations. The data collection process was conducted in the classroom environment after class hours, under the supervision of the researcher, and in accordance with the principle of anonymity. Participants were informed about the purpose of the research; it was stated that the data would be used solely for scientific purposes and voluntary participation was emphasized. Missing data accounted for less than 5% of responses and were handled using listwise deletion. The quantitative dimension of the participants was determined using G*Power analysis. It should be noted that the data in this study were obtained through self-reporting, which may cause respondent bias, such as social desirability and a tendency to present oneself in a positive light. This may limit the findings of the study.

### 2.3 Data collection tools

#### 2.3.1 International physical activity questionnaire (IPAQ)-short form

The measurement tool used in the study is a questionnaire consisting of seven items, developed to assess the relationship between individuals’ health status and physical activity levels. The questionnaire was first designed in 1996 by Austrian researcher Dr. Michael Booth and later developed into two versions, short and long by the International Physical Activity Assessment Group (IPAAG) under the name International Physical Activity Questionnaire (IPAQ). The reliability and validity of the IPAQ have been confirmed through applications conducted using the test–retest method in a total of 14 research centers across 12 countries and six different continents. The results have shown that the scale is a reliable measurement tool at the international level (Atienza, 2001). A validity and reliability study conducted on a Turkish sample was carried out among university students and Öztürk (2005) reported that the IPAQ is psychometrically adequate. The form consists of questions covering low, moderate, and high levels of activity and sitting time. The participant’s MET (Metabolic Equivalent) value is calculated by multiplying the time by 8.0 for questions related to high activity, 4.0 for questions about moderate activity, and 3.3 for questions related to low activity. The sitting question is only taken for average values and does not contribute to the total degree. It is stated that an individual’s physical activity level is considered low if it is 600 or below, medium if it is 601–3,000, and high if it is 3,001 or above. It is also seen that as the MET value increases, the individual’s physical activity level increases. However, it is not easy to meet the normality assumptions in measurements with such wide ranges. Therefore, it is stated that it is appropriate to normalize the data using some transformation techniques in multivariate data analysis. For this reason, after calculating the physical activity level in the study, a Log (10) transformation was performed during the data analysis stage. Logarithmic Transformation (Log10) is used to normalize the data, stabilizing the variance when the variance of X increases as X increases. It also normalizes the distribution of X when the data is right-skewed and linearizes the model when the dependent and independent variables exhibit a continuously increasing trend (Kalaycı, 2018).

#### 2.3.2 Life satisfaction scale

Developed to measure life satisfaction, this scale was first created by Diener et al. (1985) and its Turkish adaptation was carried out by Dağlı and Baysal (2016). The scale has a unidimensional structure and consists of five items. High scores obtained by participants on the scale indicate a high level of life satisfaction. The total score on the scale ranges from 5 to 25, with each item rated on a 5-point Likert scale (1 = Strongly Disagree, 5 = Strongly Agree). In the Turkish adaptation study, the scale was found to have high internal consistency with a Cronbach’s alpha coefficient of 0.88.

#### 2.3.3 Psychological resilience scale-short form

Developed by Smith et al. (2008) to assess individuals’ psychological characteristics, this scale was adapted into Turkish by Doğan (2015). The scale has a unidimensional structure and consists of a total of six items. Items 2, 4 and 6 of the scale are scored in the opposite direction. In the reliability analysis conducted as part of the Turkish adaptation study, Cronbach’s alpha internal consistency coefficient was calculated to be 0.83 indicating that the scale has sufficient internal consistency.

#### 2.3.4 Mindfulness scale

The scale developed by Brown and Ryan (2003) was created to measure individuals’ levels of mindfulness. The validity and reliability study of the Turkish version of the scale was conducted by Özyeşil et al. (2011). The measurement tool which has a one-dimensional structure consists of a total of 15 items. The Cronbach’s alpha internal consistency coefficient obtained during the Turkish adaptation process was found to be 0.80 thus demonstrating that the scale’s reliability is at an adequate level.

First-order confirmatory factor analyses (CFA) were conducted to assess the construct validity of the scales used in the study. Since the data met the assumption of multivariate normality, the Maximum Likelihood (ML) estimation method was used in the analyses. In the CFA results, modifications were made to the model according to the theoretical framework when necessary to achieve the fit indices accepted in the literature. Three modifications were made to enhance psychological resilience (e2 → e4, e2 → e6, e4 → e6) and three modifications were made to promote mindfulness (e2 → e3, e4 → e5, e9 → e10). The model fit, reliability levels and validity indicators of the scales used in the study were thoroughly analyzed. The threshold values proposed by Byrne (2016) and Gürbüz (2021) were used as the basis for the fit indices. The model fit indices: including the Chi-square (χ^2^) and Chi-square/degrees of freedom ratio (χ^2^/df < 5), Comparative Fit Index (CFI > 0.90–0.95), Standardized Root Mean Square Residual (SRMR < 0.08–0.05), Goodness of Fit Index (GFI > 0.90–0.95), Root Mean Square Error of Approximation (RMSEA < 0.08–0.05) (Table 1). Also, all item factor loadings were found of >0.50, indicating satisfactory construct validity (*p* < 0.001). The reliability of the scales was evaluated using Cronbach’s alpha coefficient; George and Mallery (2019) classify alpha values above 0.80 as “good” and those above 0.90 as “excellent.” According to Kalaycı (2018), values between 0.60 and 0.80 indicate acceptable reliability. In assessing construct validity, the average explained variance (AVE) and composite reliability (CR) values were also considered. Fornell and Larcker (1981) state that the AVE value should be above 0.50 and the CR value above 0.70. However, Maruf et al. (2021) state that as long as the CR value is at an appropriate level, an AVE value above 0.40 is also acceptable. All findings related to these values along with the CFA results, skewness and kurtosis values for the relevant scales are presented in a table.

**Table 1:** Goodness of fit indices and threshold values used in SEM, Cronbach alpha, normality analyses, AVE and CR.

Index	Good fit	Acceptable	PA	LS	BR	MA
χ^2^/df	<3	<3(χ^2^/df) < 5	–	1.900	4.782	3.071
GFI	>0.95	>0.90	–	0.990	0.975	0.909
CFI	>0.95	>0.90	–	0.992	0.961	0.902
RMSEA	<0.05	<0.08	–	0.050	0.072	0.076
SRMR	<0.05	<0.08	–	0.023	0.056	0.053
Cronbach Alpha	0.90	0.60–0.80	–	0.808	0.700	0.877
Skewness	–	−3/+3	−0.899	0.036	0.909	0.298
Kurtosis	–	−3/+3	0.768	−0.155	2.276	0.369
AVE	–	–		0.467	0.497	0.433
CR	–	–		0.813	0.854	0.877

PA, Physical Activity; LS, Life Satisfaction; PR, Psychological Resilience; MF, Mindfulness; X, Mean; Ss, Standard Deviation; χ^2^/df, Chi square/Degree of Freedom; GFI, Goodness of Fit Index; CFI, Comparative Fit Index; SRMR, Standardized Root Mean Square Error; RMSEA, Root Mean Square Error of Approximation; AVE, Average Variance Extracted; CR, Composite Reliability; *p < 0.05; **p < 0.01.

### 2.4 Ethical approval and data collection procedures

The data for this study were collected after obtaining informed consent from participants, as approved by the Erzincan Binali Yıldırım University Human Research Health and Sports Sciences Ethics Committee, dated January 31, 2025, with reference number E-88012460-050.04-427950 and protocol number 01/07. Participants were informed that they could withdraw from the study at any time. All procedures conducted in this study comply with the 1964 Declaration of Helsinki and relevant ethical standards. Research data were collected from participants in a face-to-face physical setting.

### 2.5 Statistical analysis

Before proceeding with data analysis missing values and outliers in the dataset were examined using SPSS 25.0 software. At this stage, the suitability of the data for the multivariate normality assumption was evaluated and the relationships between independent, dependent, mediator and moderator variables were analyzed using Pearson correlation analysis. Due to the wide range of values in physical activity form data a log10 transformation was applied to normalize the distribution. The assumption of normal distribution was tested based on Mahalanobis distances, Z scores and skewness and kurtosis values calculated for the scale scores. Skewness and kurtosis coefficients within the ±3 range along with Z values between −3 and +3, indicate that the data are normally distributed (Kalaycı, 2018). Linear relationships between variables were examined using scatter plots and no significant deviation was detected in the analysis conducted to determine whether there was a problem of multiple linear correlation. Tolerance values > 0.20 and VIF values < 10 indicated that there was no multicollinearity between the independent variables. Confirmatory Factor Analysis (CFA) was performed using AMOS 21 software to test the factor structures of the measurement tools used in the study. Following the validation of the factor structures regression analyses based on the bootstrap method were conducted to test the causal relationships between the variables (Hayes, 2018; Hayes, 2022). The PROCESS Macro, developed by Hayes (2022) was used in the hypothesis tests. Model 4 was applied to examine mediating effects, Model 1 to investigate moderating effects and Model 7 to assess situational mediating effects. Within the scope of regression analyses, the bootstrap technique was preferred utilizing 5,000 samples through resampling (Hayes et al., 2017). For findings related to the mediating effect to be considered significant, the values obtained at the 95% confidence interval (CI) must not include zero (Preacher and Hayes, 2008; Hayes et al., 2017).

## 3 Results

The correlations, means and descriptive statistics between PA, LS, PR and MF among students in the faculty of sports sciences are presented in Table 2.

**Table 2:** Correlations between variables and descriptive analyses.

Variables	PA	LS	PR	MF	X	SD
PA	1				3.58	0.356
LS	0.150**	1			16.28	4.037
BR	0.170**	0.337**	1		18.82	3.451
MA	0.170**	0.131*	0.563**	1	50.08	13.094

PA, Physical Activity; LS, Life Satisfaction; PR, Psychological Resilience; MF, Mindfulness; X, Mean; SD, Standard Deviation; *p < 0.05; **p < 0.01. The mean PA score (M, 3.58) represents log10-transformed MET values; not raw minutes. When back-transformed; this corresponds approximately to 3,800 MET-minutes per week, which falls within the “high physical activity” category.

When the table is examined, statistically significant relationships are observed between PA, LS, PR and MF. The analysis revealed low-level positive correlations between PA and LS (*r* = 0.150, *p* < 0.001), PA and PR (*r* = 0.170, *p* < 0.001) and PR and MF (*r* = 0.170, *p* < 0.001) a medium-level correlation between LS and PR (*r* = 0.337, *p* < 0.001), LS and MF (*r* = 0.131, *p* < 0.05), and PR and MF (*r* = 0.563, *p* < 0.001).

To test the first hypothesis of the study, Model 4 (PA → PR → LS) based on the mediating effect was analyzed. As a result of the bootstrap analyses, it was determined that the indirect effect of physical activity on life satisfaction through psychological resilience was statistically significant (*b* = 0.619, 95% CI [0.2185, 1.1222]). Although the indirect effect was significant, the effect size was small according to Cohen’s benchmarks, indicating that physical activity explains only a modest portion of variance in life satisfaction through resilience. It was observed that the variables included in the regression model explained approximately 12% of the total variance in life satisfaction. These results support the first hypothesis (H1).

**Figure 2:**
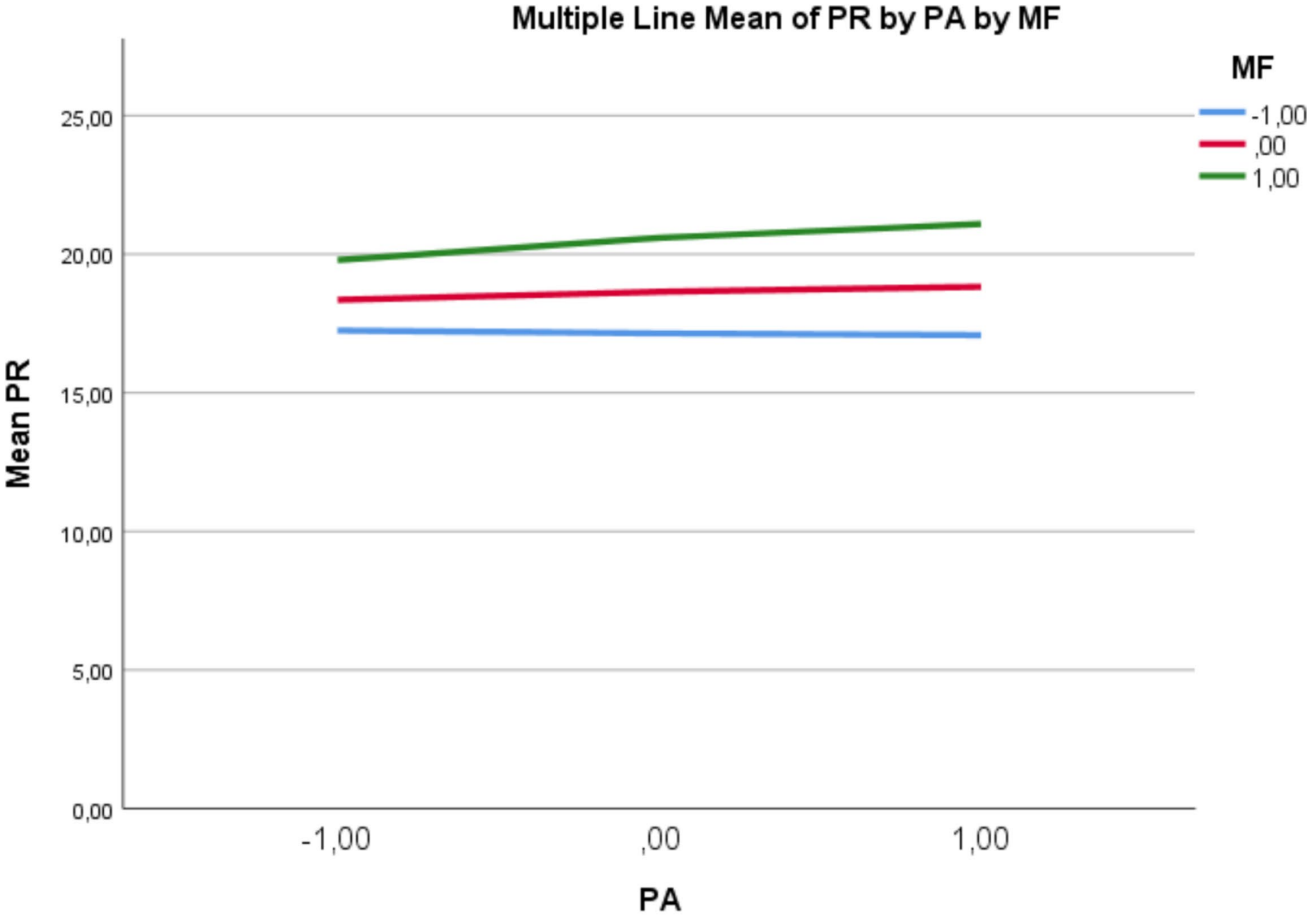
The moderating role of mindfulness in the effect of physical activity on psychological resilience.

To test the second hypothesis, a regression analysis based on Model 1, which focuses on the moderating effect was conducted. According to the results, the direct effect of physical activity on psychological resilience was not statistically significant (*b* = 0.822, *p* > 0.05). In contrast, mindfulness (*b* = 0.142, *p* < 0.01) and the interaction term Int_1 (*b* = 0.097, 95% CI [0.033, 0.160]) representing the interaction between physical activity and mindfulness yielded significant results. This suggests that mindful awareness moderates the relationship between physical activity and psychological well-being acting as a moderating variable. According to the slope analysis conducted to examine the direction and strength of the moderating effect in more detail, the effect of physical activity on resilience was not significant at low (*b* = −0.254, 95% CI [−1.311, 0.803]) or moderate (*b* = 0.716, 95% CI [−0.113, 1.547]) levels of mindfulness. However, when mindfulness was high, the effect became significant and positive (*b* = 1.979, 95% CI [0.819, 3.138]). This threshold pattern suggests that mindfulness acts as a necessary condition for physical activity to translate into resilience benefits. These findings support the second hypothesis (H2). A graphical representation of the findings is provided in Figure 2.

To test the third hypothesis of the study, a model (Model 7) was constructed in which mindfulness served as a moderated mediator. In this context, the results of the moderated mediation analysis are presented in Table 3. First, the effect of physical activity on psychological resilience was examined at different levels of mindfulness (low, medium, high). Considering the 95% confidence intervals obtained with the bootstrap method, it was found that the effect of physical activity on psychological resilience was not statistically significant when mindfulness was low (*b* = −0.254, 95% CI [−1.311, 0.803]). Similarly, the effect is not substantial for moderate mindfulness (*b* = 0.716, 95% CI [−0.113, 1.547]). However, in the case of high mindfulness, physical activity was found to have a significant and positive effect on psychological well-being (*b* = 1.979, 95% CI [0.819, 3.138]).

**Table 3:** Bootstrap regression analysis.

Variables	PR			MF			LS		
	*b*	LLCI	ULCI	*b*	LLCI	ULCI	*b*	LLCI	ULCI
Model 4 (H1)									
PA (X)	**1.650** ^**^	0.662	2.639	–	–	–	1.077	−0.039	2.195
PR (M)	–	–	–	–	–	–	**0.375** ^**^	0.260	0.490
*R* ^2^	0.029						0.122		
Model 1 (H2)									
PA (X)	0.822	−0.009	1.654	–	–	–	–	–	–
MF (W)	0.142**	0.120	0.165	–	–	–	–	–	–
X*W (Interaction)	0.097**	0.033	0.160	–	–	–	–	–	–
*R* ^2^	0.338						–		
Model 7 (H3)									
PA (X)	–	–	–	–	–	–	1.077	−0.039	2.195
PR (M)	–	–	–	–	–	–	0.375^**^	0.260	0.490
MF (W)	–	–	–	–	–	–	0.174^**^	0.085	0.264
X*W (Interaction)	–	–	–	–	–	–	**0.097** ^*^	0.033	0.160
*R* ^2^							0.338		
Moderated mediation index	**0.036** ^*^	0.007	0.072						

PA, Physical Activity; LS, Life Satisfaction; PR, Psychological Resilience; MF, Mindfulness; *b*, Unstandardized Regression Coefficient; LLCI, Lower Level Confidence Interval; ULCI, Upper Level Confidence Interval; X, Independent Variable (PA); M, Mediator Variable (PR); W, Moderator Variable (MF); Y, Dependent Variable (LS); X*W, Interaction Term (PA*MF); *R*^2^, Coefficient of Determination. The bold values indicate *p < 0.05; **p < 0.01.

In the second stage, it was examined whether the indirect effect of physical activity on life satisfaction mediated by psychological resilience was moderated by mindfulness. The significant situational mediation index (*b* = 0.036, 95% CI [0.007, 0.072]) revealed that mindfulness played a moderating role in this indirect effect. Although statistically significant, this represents a small conditional effect, becoming practically relevant only at high levels of mindfulness. This finding supports the third hypothesis (H3) of the study. According to the results of the slope analysis, the indirect effect of physical activity on life satisfaction through psychological resilience is not statistically significant when mindfulness is low (*b* = −0.095, 95% CI [−0.534, 0.295]). Similarly, no significant effect was observed at the moderate level (*b* = 0.269, 95% CI [−0.005, 0.582]). However, at the high level of mindfulness, the indirect effect of physical activity on life satisfaction through psychological resilience was found to be significant and positive (*b* = 0.742, 95% CI [0.254, 1.345]).

**Figure 3:**
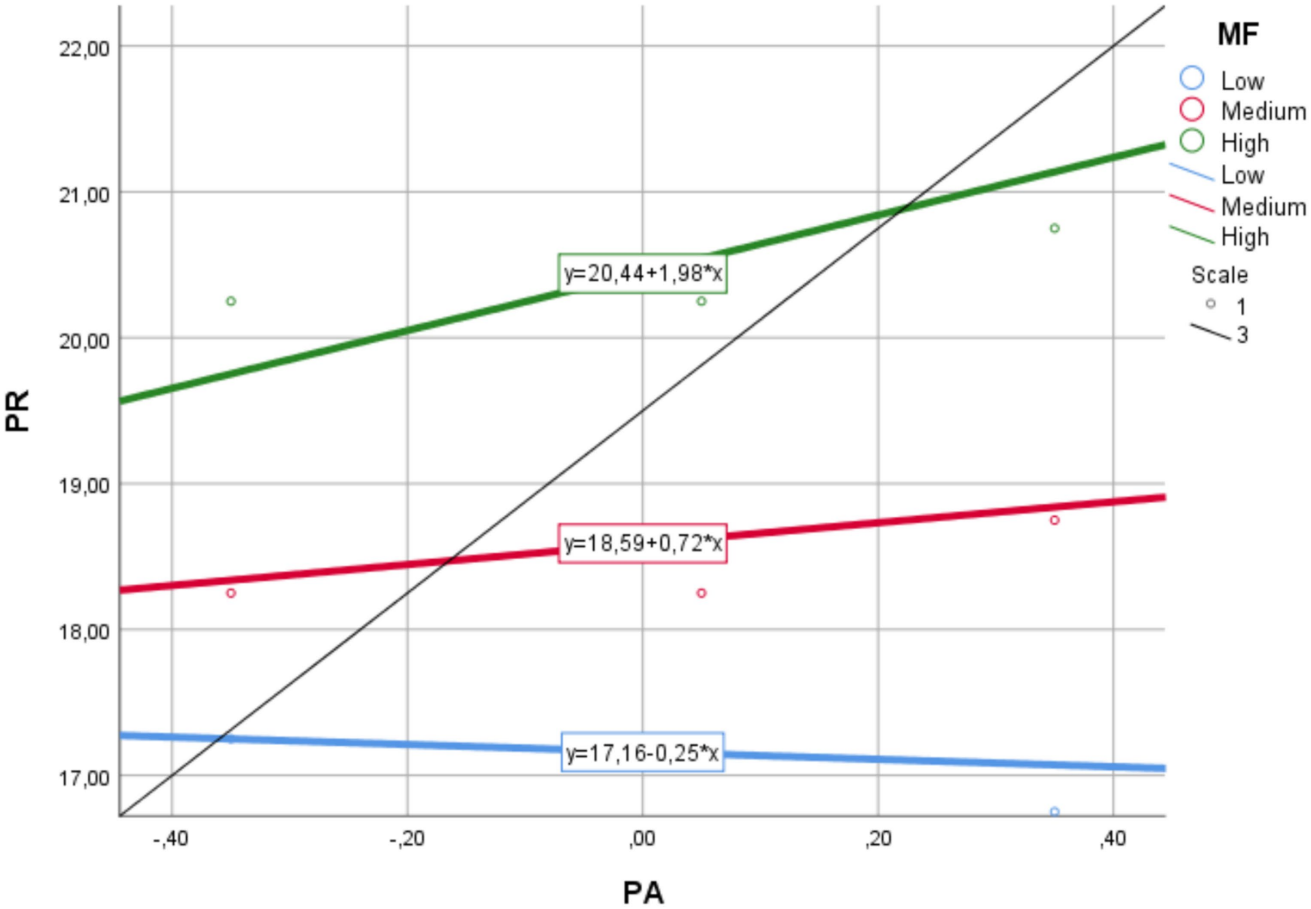
Slope analysis results.

In conclusion, it was determined that the indirect effect of physical activity on life satisfaction through psychological resilience in sports science students was significant and stronger only in cases of high mindfulness. This emphasizes that mindfulness serves as a critical moderator, enabling individuals to derive psychological and emotional benefits from physical activity only when it is sufficiently developed. The results of the slope analysis are presented in Figure 3.

## 4 Discussion

This study examined the effect of physical activity on life satisfaction focusing on the mediating role of psychological resilience and the moderating role of mindfulness. The results indicate that the contribution of physical activity is explained primarily through internal psychological resources rather than direct effects. In particular, resilience emerged as a key mechanism enhancing life satisfaction, consistent with prior research (Mandolesi et al., 2018; Ryff and Singer, 2003; Tugade and Fredrickson, 2004). Mindfulness was also identified as a crucial regulator of this process, strengthening the beneficial effect of physical activity on resilience.

The moderating role of mindfulness indicates that not all individuals benefit equally from physical activity. Students with higher mindfulness levels gained greater resilience benefits which in turn strengthened their life satisfaction. This finding is in line with prior evidence showing that mindfulness enhances stress regulation and emotional balance (Creswell, 2017; Gu et al., 2015; Lindsay and Creswell, 2017). It also supports constructivist perspectives emphasizing the importance of individual differences in adaptive processes (Langer, 2014; Shapiro et al., 2006).

The current findings are consistent with studies demonstrating that resilience is a fundamental predictor of psychological well-being and life satisfaction (Aras, 2025; Fletcher and Sarkar, 2013; Smith et al., 2008). Similarly, physical activity has been shown to promote both resilience and overall quality of life (Lines et al., 2020; Maher et al., 2015; Jevdjevic et al., 2025; Luna-Lupercio et al., 2025; Maugeri et al., 2020; Yıldırım et al., 2024). These parallels reinforce the robustness of the present findings. Eren (2023) also reported a positive interaction between physical activity, resilience, and life satisfaction, although differences in sample scope may explain variations in effect size. Similarly, Düzen and Özçelik, 2022 found that resilience predicted life satisfaction among athletes. Nie et al. (2025), however, identified emotion regulation as an additional mediator, suggesting that methodological and cultural differences may lead to variations across studies. This comparison highlights the importance of contextual factors in interpreting results.

Psychophysiological models based on mind–body unity suggest that physical activity may contribute to stress regulation, neuroplasticity, and emotion regulation, thereby supporting resilience (de Sousa Fernandes et al., 2020; Garland et al., 2015a, 2015b; Mansoor et al., 2025; McEwen, 2017; Yuan et al., 2025). Neuroscientific findings also highlight that brain mechanisms such as microglial activation and fear regulation play a role in emotional well-being, which may underlie the psychological benefits of physical activity (Zou et al., 2024). However, as biological indicators were not directly measured in this study, such explanations should be considered theoretical. From a social cognitive perspective, physical activity may increase life satisfaction indirectly by enhancing resilience through self-efficacy, problem-solving skills, and personal control (Bandura, 1986; Satici et al., 2023).

Previous research also shows that physical activity reduces anxiety and depression, while promoting positive resources that indirectly contribute to life satisfaction (Lubans et al., 2016; White et al., 2017). The present findings are consistent with this multidimensional view, underscoring that both physical and psychological resources interact to enhance well-being. Beyond resilience and mindfulness, other psychological constructs such as emotional and spiritual intelligence have also been identified as mediators of well-being and creativity (Sapiee et al., 2024). Workplace and social dynamics, including exclusion and interpersonal strategies, also influence life satisfaction and should be considered as socio-psychological mechanisms (Zhang et al., 2025).

Nevertheless, the explained variance in life satisfaction was relatively modest (*R*^2^ = 0.12), suggesting that while meaningful, these variables represent only part of the broader set of influences on life satisfaction, which also include economic, social, academic, personality, and environmental factors. In addition, stress, depression and emotional states have been shown to strongly affect subjective comfort and perceived quality of life, further underscoring the multidimensional nature of life satisfaction (Lin and Zhang, 2025). Framing the effect of physical activity on life satisfaction as an indirect process shaped by resilience, and moderated by mindfulness, therefore provides a holistic yet cautious model aligned with positive psychology and health psychology (Fredrickson, 2001; Ryff and Singer, 2008). The moderating role of mindfulness indicates that not all individuals benefit equally from physical activity. Students with higher mindfulness levels gained greater resilience benefits, which in turn strengthened their life satisfaction. This finding is consistent with prior research showing that mindfulness enhances stress regulation, emotional balance, and adaptive coping (Creswell, 2017; Gu et al., 2015; Lindsay and Creswell, 2017). In this context, incorporating mindfulness-based practices into physical activity programs may represent a promising strategy for supporting resilience and psychological well-being in university populations.

In conclusion, the findings provide a theoretical framework explaining how physical activity contributes to life satisfaction through resilience and mindfulness. These results underline the importance of developing intervention programs that incorporate resilience-building and mindfulness-based strategies, particularly for university students, who represent a developmentally sensitive group (Hülsheger et al., 2013; Röcke et al., 2009).

According to regression analysis, the model explains approximately 12% of the variance in life satisfaction indicating that variables such as physical activity and psychological resilience have a limited effect on life satisfaction. This situation can be attributed to the multidimensional nature of life satisfaction which is also influenced by factors such as economic status, social relationships, academic achievement, personality traits, stress levels and environmental conditions.

## 5 Strengths, limitations and future directions

### 5.1 Strengths

One of the most significant strengths of this study is that it examines the multilevel relationships between physical activity, psychological resilience, mindfulness and life satisfaction in the context of current psychological theories and models. Using advanced statistical approaches such as mediation and moderation models, the study not only revealed the direct relationships between variables but also detailed how the underlying psychological processes shaping these relationships are formed. Thus, the theoretical frameworks underlying the study such as Stress Coping Theory, Positive Psychology Approach and Emotional Regulation Models have been supported and deepened by the findings. In particular, the mediating role of psychological resilience and the moderating function of mindfulness were revealed aligning with the mechanisms emphasized in recent studies in these areas. Thus, the positive effects of individuals increasing their psychological resilience through physical activity on life satisfaction were strongly supported from both theoretical and practical perspectives. The methodological aspect of the study is also noteworthy. The psychometric properties of the measurement tools were carefully evaluated and validity and reliability were ensured through confirmatory factor analysis. The sample size was determined based on power analyses and the demographic diversity of the participants increased the representativeness of the results. In addition, the face-to-face collection of data and the controlled environment in which the study was conducted were essential factors that improved data quality and the reliability of the analyses. In conclusion, this study provides a unique and up-to-date contribution to the psychological health of young adults by theoretically and methodologically grounding the relationship between psychological processes and physical activity in the field of sports science. The findings obtained have the potential to shed light on both theoretical development in academic literature and the formulation of policies related to athlete health and general health.

### 5.2 Limitations

This study was conducted with a sample consisting of a limited number of sports science students, specifically within the Turkish context. This limits the generalizability of the findings to other cultural and structural contexts. The higher education system in Türkiye and the socio-cultural factors affecting young people’s participation in physical activity and psychological resilience may differ from those of student groups in other countries. Furthermore, collecting data through self-report questionnaires increases the risk of social desirability bias, and the lack of an objective measure of physical activity along with reliance on log-transformed MET values may further restrict interpretability of the results. The fact that the number of male participants (*n* = 240) in the sample is significantly higher than the number of female participants (*n* = 123) makes it challenging to assess gender-based differences accurately, which is considered a factor limiting internal validity. In addition, because participation was voluntary, recruitment bias may have influenced the composition of the sample. From a theoretical perspective, the correlational research model is insufficient to explain causal relationships between variables fully. Changes over time in variables such as psychological resilience and mindfulness could not be observed in this design. Future research should be conducted using larger and more diverse samples that cover different cultural contexts, employing objective measurement tools, experimental interventions, and longitudinal designs.

### 5.3 Future directions

The findings of this study reveal that the effect of physical activity on life satisfaction is shaped through psychological resilience and mindfulness. In future studies, the importance of mixed and experimental research methods will increase for a deeper understanding of these interactions. In particular, collecting rich data on individuals’ experiences using qualitative methods in addition to quantitative data will significantly contribute to uncovering the mechanisms of relational models. For educators, examining the interaction between students’ physical activity habits and their levels of psychological resilience and mindfulness is critical for individualizing educational programs and designing effective interventions. Policy makers and program developers should consider such multidimensional models when developing strategies to improve the physical and psychological health of the young population. In this context, the impact of physical activity on not only physical health but also psychological resilience and life satisfaction should be a fundamental goal of policies and programs. Experimental research will enable the determination of causal relationships by measuring the effects of physical activity interventions on mindfulness and psychological resilience under controlled conditions. This will clarify which types of physical activities are more effective in groups with which individual characteristics. Furthermore, the role of cultural and environmental factors in these relationships warrants further investigation. Comprehensive programs should be developed, taking this diversity into account, especially for interventions to be implemented at the local and national levels. In conclusion, mixed and experimental studies conducted with multidisciplinary approaches will serve as a bridge in translating theoretical knowledge in the field of sports science into practice. This will provide unique and lasting contributions to both academic literature and application areas.

## 6 Conclusion

This study examined the effect of physical activity levels on life satisfaction among university students in the field of sports science in the context of psychological resilience and mindfulness. The findings show that physical activity not only directly increases life satisfaction but also has indirect effects through psychological resilience. In addition, it was determined that mindfulness plays a crucial moderating role in this process enhancing the effect of physical activity on psychological resilience and consequently its impact on life satisfaction. The results of the study reveal that improving individuals’ physical activity habits, as well as increasing their levels of psychological resilience and mindfulness, contributes to their quality of life. These findings provide important clues for designing intervention programs in the fields of sports science and psychology emphasizing the need to consider psychological factors in addition to physical activity. In practical terms, the findings suggest that resilience-building and mindfulness-based modules could be incorporated into university curricula and physical education programs. Psychological counselors may also integrate physical activity into intervention strategies aimed at stress management and resilience development. For policymakers, supporting community and institutional initiatives that make structured physical activity more accessible for young adults could enhance both psychological and physical well-being. In conclusion, this study contributes to the understanding of the complex relationships between physical activity, psychological resilience and mindfulness while providing a solid theoretical and methodological foundation for future research. For educators, policymakers and program developers it is essential to develop multidimensional strategies that aim to enhance individuals’ psychological and emotional well-being.

## Data Availability

The raw data supporting the conclusions of this article will be made available by the authors, without undue reservation.
